# A Novel Helper Phage Enabling Construction of Genome-Scale ORF-Enriched Phage Display Libraries

**DOI:** 10.1371/journal.pone.0075212

**Published:** 2013-09-27

**Authors:** Amita Gupta, Nimisha Shrivastava, Payal Grover, Ajay Singh, Kapil Mathur, Vaishali Verma, Charanpreet Kaur, Vijay K. Chaudhary

**Affiliations:** 1 Department of Microbiology, University of Delhi South Campus, Benito Juarez Road, New Delhi, India; 2 Department of Biochemistry, University of Delhi South Campus, Benito Juarez Road, New Delhi, India; Federal University of Pelotas, Brazil

## Abstract

Phagemid-based expression of cloned genes fused to the *gIIIP* coding sequence and rescue using helper phages, such as VCSM13, has been used extensively for constructing large antibody phage display libraries. However, for randomly primed cDNA and gene fragment libraries, this system encounters reading frame problems wherein only one of 18 phages display the translated foreign peptide/protein fused to phagemid-encoded gIIIP. The elimination of phages carrying out-of-frame inserts is vital in order to improve the quality of phage display libraries. In this study, we designed a novel helper phage, AGM13, which carries trypsin-sensitive sites within the linker regions of gIIIP. This renders the phage highly sensitive to trypsin digestion, which abolishes its infectivity. For open reading frame (ORF) selection, the phagemid-borne phages are rescued using AGM13, so that clones with in-frame inserts express fusion proteins with phagemid-encoded trypsin-resistant gIIIP, which becomes incorporated into the phages along with a few copies of AGM13-encoded trypsin-sensitive gIIIP. In contrast, clones with out-of-frame inserts produce phages carrying only AGM13-encoded trypsin-sensitive gIIIP. Trypsin treatment of the phage population renders the phages with out-of-frame inserts non-infectious, whereas phages carrying in-frame inserts remain fully infectious and can hence be enriched by infection. This strategy was applied efficiently at a genome scale to generate an ORF-enriched whole genome fragment library from *Mycobacterium tuberculosis,* in which nearly 100% of the clones carried in-frame inserts after selection. The ORF-enriched libraries were successfully used for identification of linear and conformational epitopes for monoclonal antibodies specific to mycobacterial proteins.

## Introduction

Phage display is a powerful technique for studying protein-ligand interactions and identification of immunodominant regions using gene fragment libraries. In addition, it has been exploited for epitope mapping and construction of large antibody libraries to select desired binders with improved affinities [1], [2].

Among different phage display systems, gIIIP of the filamentous bacteriophage M13 is most widely employed. The gIIIP is a 406 amino acid protein with a maximum of five copies per phage. It comprises three functionally distinct domains: N1, N2 and CT, which are separated by glycine rich linkers [3]. These domains play a crucial role in infection and phage assembly; however, peptides and proteins can be inserted at the boundaries between the gIIIP domains without affecting the infectivity of the phage [4], [5]. For gIIIP-based display, vectors based on phagemid carry the gene encoding *gIIIP* under the control of a regulated promoter, with the foreign DNA cloned between a signal sequence and the *gIIIP* coding sequence [6]–[8]. Phage production is initiated by infection with a helper phage (such as VCSM13), which provides all of the proteins necessary for the replication and assembly of phage particles. The extruded phage particles encapsulate phagemid single-stranded DNA and display two types of gIIIP protein: one encoded by the helper phage (native gIIIP protein) and the other encoded by the phagemid (gIIIP fusion protein).

The use of phage display technology in constructing cDNA libraries has been challenging due to the stop codons and the polyA tail present in full-length mRNA [9], [10]. Using randomly primed cDNA fragments can alleviate this limitation, however, the majority of clones remains out-of-frame (17 out of 18 possible frames). This problem is also encountered in gene fragment libraries made from random fragments of gene/genome sequences. Also, during the construction of complex antibody libraries, PCR is employed at multiple steps. PCR generated errors result in a large number of cloned antibody fragments either having stop codons or out of frame mutations, thus reducing the quality of the libraries. Consequently, large libraries with several million to billion clones are constructed; however, the effective functional population of in-frame clones in these libraries is only 5–6%. Further, when used for affinity selection, these libraries suffer from non-specific interactions leading to poor enrichment of desired clones [11]. The success of selection of specific interactions can be remarkably increased if the quality of input library is improved. Elimination of out-of-frame clones to enrich the libraries for ORF clones is a step in this direction.

Different systems have been developed for the selection of gene fragments in the correct reading frame and construct ORF-selected phage display libraries. In one system, the gene fragments are cloned between the signal sequence and the coding sequence of β-lactamase, so that only in-frame fragments result in expression of functional β-lactamase to impart ampicillin resistance [12]. However, after selection, these putative in-frame fragments need to be transferred to a phage display vector by cloning [11] or the coding sequence of β-lactamase must be deleted by *Cre*-*Lox* recombination [12], [13] to produce a signal sequence in-frame with *gIIIP* for display of the cloned gene fragments. In another system, hyperphage (a helper phage with *ΔgIII*) has also been used to enrich clones which carry in-frame DNA inserts [14]. However, the phage titres produced by hyperphage are much lower than those produced using VCSM13 helper phage. Furthermore, when applied to the selection of ORFs in a genome scale library, 69% of the clones were in-frame, but the average size distribution of the selected library decreased to 50–300 bp, compared to 50–700 bp for the originally cloned DNA fragments [15].

In this paper, we describe the design of a novel helper phage, AGM13, which carries two copies of trypsin cleavage sites in the linker domains of gIIIP. Trypsin treatment of phages carrying this variant of gIIIP cleaves the N1 and N2 domains from the CT domain, thereby abolishing the infectivity of the phages. In the context of ORF selection, AGM13 is used to rescue the phages from phagemids carrying gene fragments fused to trypsin-resistant gIIIP. Therefore, only clones which carry gene fragments in-frame with the signal sequence and *gIIIP* can display trypsin-resistant functional gIIIP fusion protein along with a few copies of helper phage-derived trypsin-sensitive gIIIP. In contrast, clones harboring out-of-frame inserts produce phages displaying only trypsin-sensitive gIIIP from AGM13. Hence, trypsin treatment of such a phage population would render all of the phages with out-of-frame inserts non-infectious, leaving the phages with in-frame inserts fully infectious and recoverable by infection.

This technique of ORF selection using the AGM13 helper phage was applied at a genome scale to generate an ORF-enriched whole genome fragment library of *Mycobacterium tuberculosis*. The library contained greater than 95% clones carrying in-frame inserts with no change in the fragment size and genome representation.

## Materials and Methods

### Materials

*Escherichia coli* strains TOP10F’ (F’ [*lacI^q^* Tn10 (*tet* ^R^)] *mcr*A Δ(*mrr-hsd*RMS-*mcr*BC) φ80*lac*ZΔM15 Δ*lac*X74 *deo*R *nup*G *rec*A1 *ara*D139 Δ(*ara-leu*)7697 *gal*U *gal*K *rps*L(*Str* ^R^) *end*A1 λ^−^) and TG1 {[F’, *tra*D36 *pro*AB^+^ *lacI* ^q^ *lac*ZΔM15] *sup*E *thi-*1 Δ(*lac-pro*AB) Δ(*mcr*B-*hsd*SM)5, (r_K_^−^m_K_^−^)]} were obtained from commercial sources (Life Technologies Corporation, Carlsbad, USA). MAb 2911 and 30421 are monoclonal antibodies produced in-house against the gVIIIp and gIIIP coat protein of M13 phage [16]. MAb Ag85-12 and MAb 1912 are monoclonal antibodies produced in-house against Ag85B (Rv1886c) and 19 KDa (Rv3763) proteins of *M. tuberculosis*. Trypsin was purchased from Sigma Chemical Co. (Sigma, St Louis, MO, USA); Sepharose CL-6B was purchased from GE Healthcare (GE-Amersham Health Science, Uppsala, Sweden). MAb1905 is a monoclonal antibody produced in-house against a 19 kDa *M. tuberculosis* antigen encoded by the gene *Rv3763*. The MPT64 antigen, a histidine-tagged recombinant protein encoded by the *M. tuberculosis* gene *Rv1980c*, was produced in *E. coli* and purified to homogeneity by affinity chromatography. In different formats, 33scFv is a single chain antibody fragments derived from genes encoding the variable domains of MAb 33 against the *M. tuberculosis* protein MPT64; the fragments were displayed fused to full-length gIIIP using a phagemid-based vector. The polyclonal antibody to M13 phage was produced by commercial service provider (Bangalore Genei Pvt. Ltd., India) and was obtained from the serum of a rabbit immunized with purified M13 phages obtained after cesium chloride density centrifugation.

### Construction of AGM13 Helper Phage

The sequence encoding a trypsin cleavage site (KDIR) was introduced into the helper phage VCSM13 (Agilent Technologies, Texas, USA) by site-directed mutagenesis using Kunkel’s method [17]. The oligonucleotide 5′-AGCCACCACCCTCAGAGCCGCCACCACGAATATCTTTAGAGCCGCCGCCGGCATTGACAGG-3′ was used to insert the codons for KDIR within the two linker regions of gIIIP. Following *in vitro* second strand synthesis, the mutagenesis mixture was electroporated in *E. coli* TG1 and plated to obtain plaques. The plaques were suspended in 100 µl NET buffer (0.15 M NaCl in 20 mM Tris pH 8.0, containing 1 mM EDTA) and used as a template for PCR to amplify the *gIIIP* coding sequence. The presence of the KDIR sequence was confirmed by sequencing the amplified product. The selected clones were grown to produce phages by infecting log-phase *E. coli* TG1 and the cell free supernatant was used for phage titration. One of the clones was cultured in a large volume to obtain phages, which were purified by PEG-NaCl precipitation and Sepharose CL-6B column chromatography as described below.

### Phage Production and Purification

To prepare AGM13 stock, *E. coli* TG1 cells were grown to a mid-log phase (OD_600_ = 0.5). Cells of 100 ul were infected with 100 µl dilution of helper phage and incubated at 37°C for 30 min. Cultures were mixed with LB soft agar and poured on LB plates. Plates were incubated overnight at 37°C. Plaques were counted to determine the number of plaque-forming units (PFUs). A plaque of AGM13 was mixed with 2 ml log phase *E. coli* TG1 cells and grown at 37°C for 30 min with no shaking followed by 1 hr at 37°C with shaking at 100 rpm. The TG1-AGM13 mixture was then diluted in 200 ml 2× YTK (2× YT medium containing 50 µg/ml kanamycin) and grown at 37°C with shaking at 200 rpm for 16 hr. The supernatant was harvested by two successive centrifugation cycles (23,434 *g* for 10 min at 4°C). The phage particles were precipitated from the supernatant by incubating with 0.15 volume of 16.67% polyethylene glycol (PEG 6000–8000)/3.3 M NaCl for 4 hr at 4°C followed by centrifugation at 23,440 *g* for 30 min at 4°C. The precipitated phages were resuspended in NET buffer and centrifuged at 26,900 *g* for 10 min at 4°C to remove the insoluble material. The phage particles in the clear supernatant were finally purified on a 10 ml Sepharose CL-6B column. The phage supernatant (3 ml) was loaded onto column equilibrated with NET buffer and flow through was discarded. NET buffer of 0.2 ml was added in the column and eluate was discarded. Finally, the phages were eluted using 3.8 ml NET buffer. The phage titre was determined as PFU by infecting log phase *E. coli* TG1. Aliquots of purified phages were stored at –20°C.

### Phage Quantification and Reactivity Determination by ELISA

The wells of 384-well MaxiSorp™ microtitre ELISA plates (Nalge Nunc Int’l, New York, USA) were coated with 25 µl of anti-gVIIIp MAb (2911 IgG), anti-gIIIP MAb (30421 IgG), MPT64 antigen or MAb 1905, all diluted to 2 µg/ml in phosphate buffered saline (PBS). After blocking with 2% non-fat dry milk, serial dilutions of purified phages rescued with either AGM13 or VCSM13 (3×10^9^−3×10^5^/ml for the MPT64 antigen and MAb 1905 plates, and 1×10^8^−1×10^5^/ml for anti-M13 MAb plates) were added to the coated wells and incubated for 1 h at room temperature. After washing, the bound phages were detected with HRP-conjugated anti-gVIIIp MAb 2911. The plates were washed, developed with 3,3′,5,5′-tetramethylbenzidine (TMB) as a substrate, and absorbance was determined at 450 nm using an ELISA plate reader (SpectraMax M5; Molecular Devices, Sunnyvale, CA, USA).

### Assay of Helper Phage Trypsin Sensitivity and Immunoblotting

Purified phage particles of AGM13 and VCSM13 (2×10^12^; as estimated by the plaque-forming units [PFU] assay) were incubated in total volume of 1.0 ml with different concentrations of trypsin (0, 0.1, 1.0, 10, 100 µg/ml) in PBS for 30 min at 37°C. The phage suspensions were immediately used for titration with *E. coli* TOP10F’ to score the number of PFU. Separately, for Western blot analysis, 1.5×10^10^ trypsin-treated phages were electrophoresed under reducing conditions on 10% SDS-PAGE, followed by electroblotting onto 0.45 µ PVDF membranes (Immobilon; Millipore, Bedford, MA, USA). After blocking, the blots were probed with mouse anti-gIIIP MAb (30421) and a rabbit polyclonal antibody against whole M13 phage, followed by HRP-conjugated goat anti-mouse and goat anti-rabbit IgG (H+L) antibodies (Jackson Immuno-Research Laboratories, West Grove, PA, USA), respectively, and the bands were visualized using 3,3′-diaminobenzidine (DAB) as a substrate.

### Phagemid Display Vector and Gene Fragment Libraries

The high copy number phagemid vector pVCEPI23964 (Fig. S1 in Data S1) has a backbone derived from pUC119 and carries (under the control of *lac*PO between *Hind*III and *Eco*RI sites) a DNA cassette comprising a *Xba*I site, ribosome binding site (RBS), pectate lyase B signal sequence (*PelBss*), 1.8 kb stuffer flanked by unique restriction sites followed by the codons for a trypsin cleavage site (KDIR) and full length *gIIIP* (2–405 codons), with appropriate spacers comprising glycine and serine residues. The gene fragments were inserted in place of the 1.8 kb stuffer using a restriction enzyme-free cloning strategy. The vector backbone further comprises the ColE1 origin of replication (Ori), the wild-type filamentous phage origin of replication (f_ori_) and the β-lactamase gene as a selection marker. The *lac*PO is preceded by a tHP transcriptional terminator from the glutamine permease operon (*glnHPQ*) of *E. coli* [4].

Two gene fragment libraries containing fragments of 100–300 bp and 300–800 bp from the *M. tuberculosis* genome were constructed by ligating three molar excess of the fragments with 2 µg of the vector pVCEPI23964, for display of the encoded proteins fused to full-length gIIIP. The entire ligated sample was electroporated in *E. coli* TOP10F’ cells (50 electroporations), regenerated in SOC medium for 1 h, and then plated on one hundred 150 mm plates containing LBAmpGlu (LB agar containing 100 µg/ml ampicillin and 1% w/v glucose). The grown cells were cultured overnight, scraped into 2× YT mixed with glycerol storage solution (65% glycerol, 0.1 M Tris pH 8.0, 25 mM MgSO_4_) and stored at −80°C labeled as C01. The library size was determined by plating a dilution of regenerated cells on LBAmpGlu plates. The libraries were named MTBLIB25 for the 100–300 bp fragments and MTBLIB27 for the 300–800 bp fragments.

### Production of Phages from Primary Transformants

An aliquot of pooled transformants (C01) containing 2.5×10^9^ cells was diluted in 200 ml of 2× YTG (2× YT containing 1% w/v glucose) and grown at 37°C with shaking at 200 rpm to an OD_600nm_ of 0.1–0.2. Ampicillin was added to a final concentration of 100 µg/ml, followed by growth at 37°C with shaking at 220 rpm until the OD_600nm_ reached 0.4–0.5. At this point, the culture was maintained with slow shaking at 100 rpm for 30 min, and then 50 ml culture (∼2.5×10^10^ cells) was infected with helper phage AGM13 at multiplicity of infection (MOI) of 20 (5.0×10^11^ phages). The culture was kept at 37°C without shaking for 30 min, followed by shaking at 100 rpm for 1 h at 37°C. The infected culture was diluted 10-fold in 2× YTAK (2× YT medium containing 100 µg/ml ampicillin and 50 µg/ml kanamycin) and grown at 32°C with shaking at 220 rpm for 16 hr. The phages were harvested from the culture supernatant, and purified by PEG-NaCl precipitation followed by column chromatography, as described above. The phage titre was determined as the number of colony-forming units (CFU) by infecting log-phase *E. coli* TOP10F’ cells. The phage libraries of 100–300 bp and 300–800 bp were named MTBLIB25P01 and MTBLIB27P01, respectively.

### Selection of Clones with Open Reading Frames by Trypsin Treatment

To optimize the trypsin treatment of primary library phages (P01), 1×10^11^ purified phage particles were diluted in PBS, incubated with 0, 10, 100 and 200 µg/ml trypsin in a total volume of 1.0 ml at 37°C for 30 min, then the phage titre was determined as the number of Amp^r^ transductants on *E. coli* TOP10F’ (Table S1.A and B). The transductants obtained were analyzed by colony PCR using the 5′ primer M13R (5′-AGCGGATAACAATTTCACACAGGA-3′) and 3′ primer U251 (5′-AGTTTTGTCGTCTTTCCAGACGT-3′). The PCR products obtained were sequenced using M13R and U251 primers by ABI PRISM dye terminator cycle sequencing ready reaction kit on Applied Biosystems 3730 automated sequencer.

For ORF selection, 1×10^12^ purified phages from the primary library (P01) were treated with 10 µg/ml trypsin in a total volume of 1.0 ml for 30 min at 37°C. Trypsin-treated phages were used to infect 1×10^9^ TOP10F’ cells (grown to OD_600nm_ 0.4–0.5) at 37°C for 30 min without shaking, followed by slow shaking at 100 rpm for 30 min at 37°C. The infected cells were pelleted by centrifugation at 1500 g at room temperature (23–25°C) for 5 min, washed twice with 5 ml of 2× YTAG (2× YT medium containing 100 µg/ml ampicillin and 1% w/v glucose) to ensure complete removal of trypsin, re-suspended in 20 ml 2× YTAG, plated on twenty 150 mm LBAmpGlu plates and grown overnight. The colonies were scraped into 30 ml 2× YT, stored at −80°C in glycerol storage solution and labeled MTBLIB25C02 and MTBLIB27C02 for ORF-selected transductants of 100–300 bp and 300–800 bp, respectively.

The phage libraries were rescued from 1.0×10^10^ cells of the ORF-selected transductants (C02) using AGM13 as described above and labeled as MTBLIB25P02 and MTBLIB27P02 for the ORF-selected phage libraries of 100–300 bp and 300–800 bp, respectively. PCR analysis and DNA sequencing were used to screen for recombinant clones. The nucleotide and protein sequences of the recombinants were analyzed using BLASTn or BLASTp (NCBI) and aligned to the *M. tuberculosis* genome.

### Affinity Selection Protocol

For panning of the whole genome-fragment libraries on MAbs, eight test wells each were coated with 2 µg/ml of MAb Ag8512 (Ag85A and Ag85B specific) or MAb 1912 (19 KDa specific) antibodies, or buffer PBS (Control wells) and incubated at 4°C overnight. After blocking with 2% PBSTB (PBS buffer containing tween 20 and 2% bovine serum albumin), purified phages (1×10^9^ phages per well of MTBLIB25 and MTBLIB27 libraries) were added to each well, incubated at 37°C for 1 h and unbound phages removed by washing. For both the libraries, the captured phages were eluted using low-pH buffer, 0.2 M glycine-HCl, pH 2.2 and determined as the number of colony-forming units (CFU) by infecting log-phase *E. coli* TOP10F’ cells. To amplify phages for the next round of panning, Top10F’ cells were infected at MOI of 0.1 with phages eluted above by incubating cells with phages at 37°C for 30 min. The infected cells were pelleted by centrifugation at 1500 g at room temperature (23–25°C) for 5 min and re-suspended in 20 ml 2× YTG. The culture was processed as described above for the production of phages from primary transformants.

For the second round of panning, 1×10^9^ amplified phages were used and panning was carried out as described above for the first round. The eluate of second panning (Pan II eluate) was used to determine titre of phages in eluate and was amplified further for the third round of panning (Pan III eluate).

## Results

### Design and Characteristics of the Trypsin-sensitive Helper Phage AGM13

**Figure 1:**
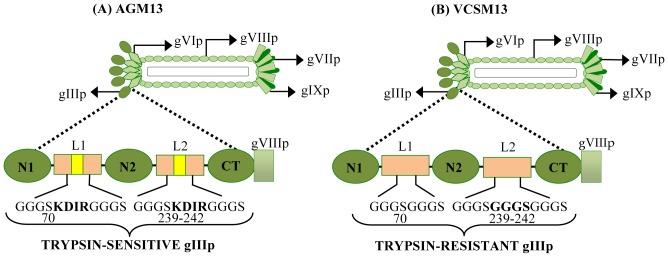
Schematic representation of gIIIP protein encoded by the helper phages AGM13 and VCSM13. The N1, N2 and CT domains of the coat protein gIIIP are depicted as oval boxes (green) and are joined by the linkers L1 and L2. (A) A four-amino acid ‘KDIR’ trypsin cleavage site was introduced into both L1 and L2 linkers in the AGM13 helper phage, to encode trypsin-sensitive gIIIP. (B) VCSM13 is the wild-type helper phage without any trypsin cleavage sites, which encodes a trypsin-resistant gIIIP. The ‘KDIR’ amino acid sequence was inserted after residue 70 of gIIIP in L1 (shown in bold in A) and replaced four amino acids between residues 239–242 of gIIIP in L2 (shown in bold in B).

**Figure 2:**
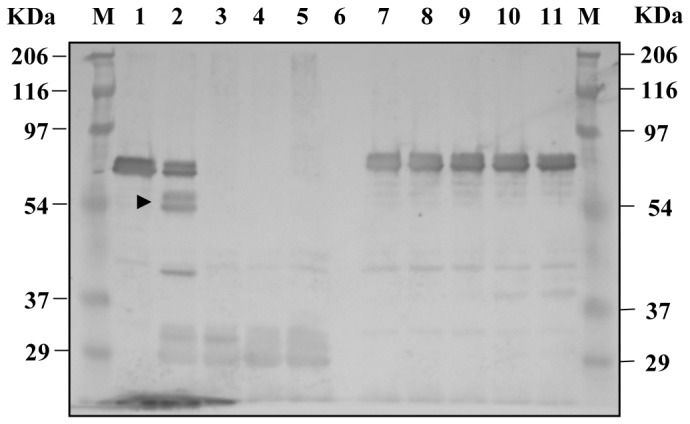
Western blot analysis of AGM13 and VCSM13 phage samples treated with different concentrations of trypsin. Aliquots equivalent to 1.5×10^10^ trypsin-untreated and -treated phages (analyzed in Table 1) were separated by 10% SDS-PAGE under reducing conditions, transferred onto 0.45 µ PVDF membranes and probed with polyclonal antibodies against bacteriophage M13. M: Prestained marker; Lanes 1–5: 1.5×10^10^ AGM13 phages treated with 0, 0.1, 1, 10 and 100 µg/ml trypsin, respectively; Lane 6: empty; Lanes 7–11: 1.5×10^10^ VCSM13 phages treated with 0, 0.1, 1, 10 and 100 µg/ml trypsin, respectively.

**Figure 3:**
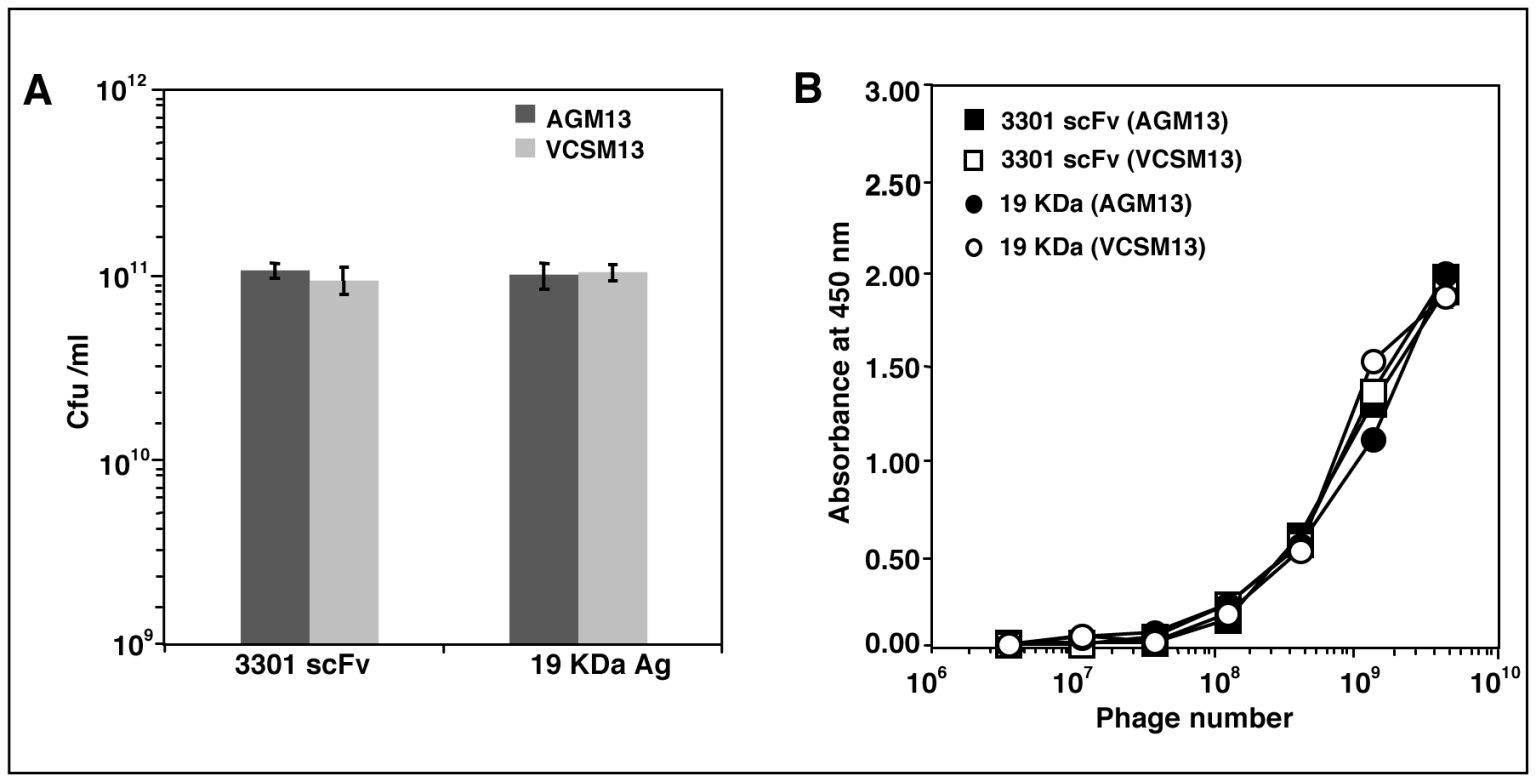
Titre and reactivity of phages rescued by AGM13 and VCSM13. (A) The phagemid clones rescued with AGM13 and VCSM13 were titrated on *E. coli* TG1 and scored as the number of Amp^r^ transductants (CFU/ml); 3301 scFv is an antibody fragment specific to the protein MPT64 encoded by the *M. tuberculosis* gene *Rv1980c*; 19 kDa antigen is encoded by the *M. tuberculosis* gene *Rv3763*; and MAb 1905 is a monoclonal antibody against the 19 kDa antigen. (B) Different dilutions of rescued phages were added to Maxisorb™ plates coated with MPT64 (for 3301 scFv-displaying phages) or MAb 1905 (for 19 kDa antigen-displaying phages). The bound phages were detected using a HRP conjugated anti-phage MAb. Values are mean ± SD of three independent experiments. For Fig. 3B error bars are too small to be visible as compared to the physical size of the symbol.

To create the helper phage AGM13 expressing gIIIP with trypsin cleavage sites (trypsin-sensitive gIIIP), the sequence encoding the four amino acids KDIR was introduced within the the two linkers (L1 and L2) between the domains of gIIIP in the phage VCSM13 by site-directed mutagenesis (Fig. 1). The four amino acid sequence was inserted after amino acid 70 in L1 between the N1 and N2 domains, and also replaced the four amino acids at 239–242 in L2 between the N2 and CT domains of wild-type gIIIP. The yield of AGM13 and VCSM13 phage particles in *E. coli* TG1 cells was comparable, with titres of 2×10^11^/ml. AGM13 phages were highly sensitive to trypsin; treatment with 0.1 µg/ml trypsin reduced the titre of infectious phages by 6 orders of magnitude, and by an additional 3 orders of magnitude at 10 µg/ml, indicating that the novel trypsin cleavage sites in gIIIP were fully exposed (Table 1). In contrast, VCSM13 remained fully infectious, even after incubation with 100 µg/ml trypsin, while AGM13 was non-infectious at this concentration (Table 1). Furthermore, Western blot analysis using polyclonal antibodies against M13 phage particles (Fig. 2) revealed that more than 50% of the gIIIP expressed by AGM13 was cleaved upon treatment with 0.1 µg/ml trypsin, releasing an approximately 50 kDa immunoreactive band. This band probably corresponds to the N2-CT fragment (Fig. 2, Lane 2), as the same band was also detected using a monoclonal antibody against gIIIP that recognizes an epitope located in the N2 domain (Fig. S2, Lane 2). At higher concentrations of trypsin, the full-length gIIIP band, as well as the N2-CT band, disappeared completely (Fig. 2, Lanes 3–6). Bioinformatic analysis identified multiple putative trypsin cleavage sites within gIIIP, which probably became exposed after initial cleavage of the KDIR sequences in the gIIIP of AGM13, resulting in total degradation of gIIIP and loss of phage infectivity. The gIIIP of VCSM13 remained unaffected even when incubated with 100 µg/ml trypsin, suggesting that the putative trypsin sites are not exposed in wild-type gIIIP (Fig. 2, Lanes 7–11). Further, phage production from a phagemid vector for gIIIP display using AGM13 was comparable to that using VCSM13, when scored as the number of ampicillin resistant (Amp^r^) transductants for two different displayed molecules, namely a single chain fragment variable (scFv) and 19 kDa protein (Fig. 3A). Moreover, using a phage ELISA to estimate the functional activity of the displayed molecules, the phages rescued by AGM13 had comparable reactivity to the phages produced by VCSM13 (Fig. 3B). These results clearly demonstrated that incorporation of the trypsin cleavage sites did not alter the “helper” properties of the phage AGM13.

**Table 1:** Trypsin sensitivity assay.

Trypsin concentration(µg/ml)	PFU/ml	
	AGM13	VCSM13
0.0	4.6×10^12^	5.4×10^12^
0.1	3.4×10^6^	3.2×10^12^
1.0	2.6×10^6^	3.0×10^12^
10.0	2.6×10^3^	2.2×10^12^
100.0	0	1.6×10^12^

Purified phages (2×10^12^) were treated with trypsin in total volume of 1.0 ml for 30 min at 37°C. The phages were titrated by infecting *E. coli* TG1 and the numbers of PFU were scored.

### AGM13 Enables Efficient Selection of ORF Bearing Phages

The rationale of the trypsin-sensitive helper phage AGM13 for ORF selection is based on the premise that once a DNA fragment is cloned in between the signal sequence and the coding sequence of trypsin-resistant *gIIIP* in a phagemid vector, trypsin-resistant gIIIP fusion proteins will only be produced by clones in which the cloned DNA fragment is in-frame with both the signal sequence and *gIIIP* (in-frame clones), since a complete fusion protein with both a signal sequence and gIIIP is required for periplasmic export and incorporation into the extruding phages. Therefore, upon phage rescue with AGM13, the in-frame phagemid clones would produce phages expressing a fusion moiety with phagemid-derived trypsin-resistant gIIIP along with some copies of trypsin-sensitive gIIIP supplied by AGM13. In contrast, clones carrying a DNA fragment which is out-of-frame with the signal sequence and *gIIIP* (out-of-frame clones) would not produce trypsin-resistant gIIIP fusion proteins and the extruding phages would incorporate only trypsin-sensitive gIIIP supplied by AGM13. When this phage population is treated with trypsin, only phages expressing trypsin-resistant gIIIP fusion proteins, with the insert in-frame with gIIIP, would remain infectious.

**Figure 4:**
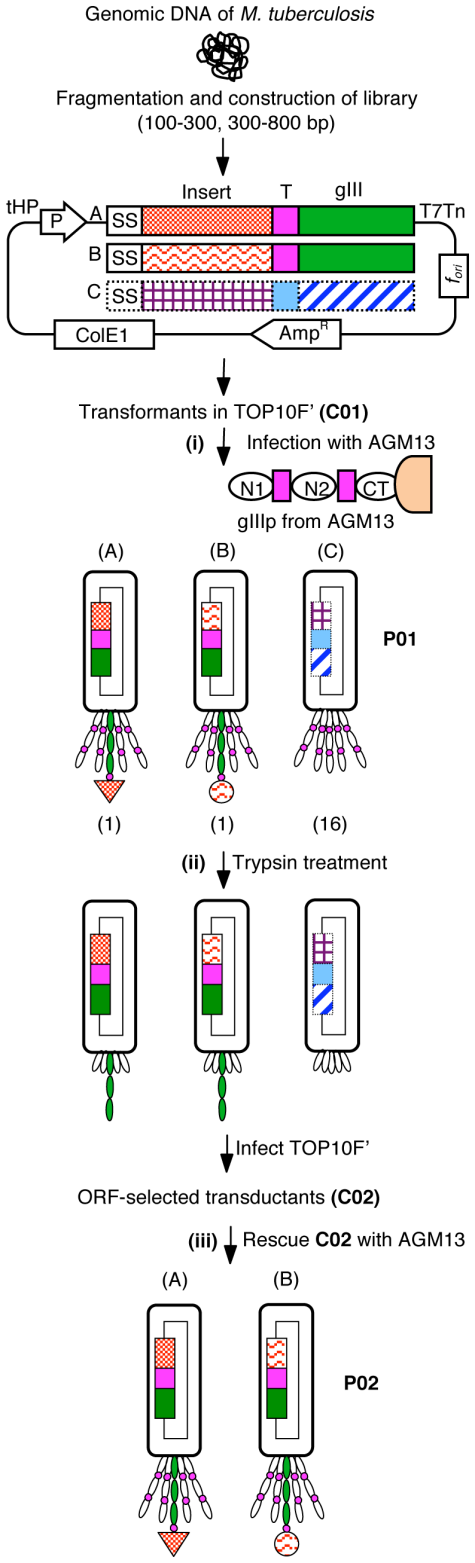
Process of open reading frame (ORF) selection using the AGM13 helper phage. The gene-fragment library is constructed in a phagemid vector by inserting the fragments between the *PelB* signal sequence and full length native *gIIIP*, and transformants (C01, primary unselected library) are obtained in *E. coli* TOP10F’. (i) The C01 cells are used for producing phages using AGM13, which has a trypsin cleavage site (T) in the linker regions between the N1 and N2, and N2 and CT domains of gIIIP. During rescue, three types of phages are produced: A, Phages displaying protein matching with the *M. tuberculosis* proteome (genic clones) expressed as fusion protein with full length trypsin-resistant gIIIP; B, phages displaying protein not matching with the *M. tuberculosis* proteome (non-genic clone) but expressed as fusion protein with full length trypsin-resistant gIIIP. C, phages not displaying any protein as the insert is out-of-frame with the signal sequence and trypsin-resistant *gIIIP*, which carry only AGM13-encoded trypsin-sensitive gIIIP. (ii) Trypsin treatment of the phage population renders non-displaying phages (type C) non-infectious, while A and B become fully infectious due to removal of the displayed protein by cleavage of the trypsin site present between gIIIP and the fusion partner. Subsequent infection of the trypsin-treated library produces ORF-selected transductants (C02). (iii) Upon rescue with AGM13 or any other helper phage, the C02 transductants produce ORF-selected phages displaying protein, which could be from genic or non-genic DNA sequences.

Based on this principle of ORF selection by AGM13 at the genome scale, two large gene fragment libraries (>0.5–1×10^8^ clones) containing 100–300 bp and 300–800 bp fragments of the *M. tuberculosis* genome were constructed by cloning randomly generated fragments in between the PelB signal sequence (*PelBss*) and full-length trypsin-resistant *gIIIP* in a phagemid vector (Fig. 4). The library of 100–300 bp fragments contained ∼5×10^7^ independent clones and was named MTBLIB25C01; the library of 300–800 bp fragments contained ∼1×10^8^ independent clones was named MTBLIB27C01 (C01 represents cells containing unselected primary clones; Fig. 4). Sequence analysis of randomly selected transformants from both libraries revealed that nearly 97% of the clones were recombinants with nucleotide sequences aligning to the *M. tuberculosis* genome (Table 2A and B, I.1). However, only 1% of the clones carried fragments in-frame with *PelBss* and *gIIIP*; these clones were predicted to produce phages bearing trypsin-resistant gIIIP fusion protein upon rescue (Table 2A and B, II.1). Accordingly, both libraries were rescued using AGM13 to obtain phage titres of approximately 1×10^11^/ml, scored as the number of Amp^r^ transductants. The primary phage libraries of 100–300 bp and 300–800 bp were named MTBLIB25P01 and MTBLIB27P01, respectively (P01 represents primary unselected phage library; Fig. 4). Screening of the clones from the libraries revealed that almost 100% of the primary phage transductants were recombinants and contained fragments aligning with the *M. tuberculosis* genome (Table 2A and B, I.2). However, similarly to C01, only 2% of the clones contained inserts, which were in-frame with *PelBss* and *gIIIP* (Table 2A and B, II.2). Conceptually, only these clones (2%) should display a fusion protein, expressing the insert in-frame with PelBss and phagemid encoded trypsin-resistant gIIIP on the phage particle. To select clones with in-frame inserts and eliminate clones with out-of-frame inserts, the purified P01 phages (∼10^12^/ml) from each library were treated with different concentrations of trypsin and used to infect *E. coli* TOP10F’ to obtain transductants (C02 represents cells of ORF-selected clones). As shown in Table S1, treatment of P01 phages with 10 µg/ml trypsin resulted in enrichment of ORF-clones by greater than 90% and no enhancement was observed with higher concentrations of trypsin. Therefore, 10 µg/ml trypsin was used for the treatment of P01 phages to obtain C02 library.

**Table 2:** Analysis of random clones at various stages of library construction.

Stage No.	Stage	I. Aligning to *M. tb* genome*	II. ORF in-frame with *PelBss* (and *gIIIP*)†	III. Aligning to *M. tb* proteome‡
**A. MTBLIB25 Library (100–300 bp)**				
1.	MTBLIB25 C01	96.8 (92/95)	1.05 (1/95)	–
2.	MTBLIB25 P01	100 (47/47)	2.1 (1/47)	–
3.	MTBLIB25 C02	100 (48/48)	89.6 (43/48)	46.5 (20/43)
4.	MTBLIB25 P02	100 (48/48)	93.7 (45/48)	55.5 (25/45)
**B. MTBLIB27 Library (300–800 bp)**				
1.	MTBLIB27 C01	97.8 (90/92)	1.1 (1/92)	100 (1/1)
2.	MTBLIB27 P01	100 (46/46)	2.2 (1/46)	–
3.	MTBLIB27 C02	100 (45/45)	95.5 (43/45)	53.5 (23/43)
4.	MTBLIB27 P02	100 (46/46)	97.8 (45/46)	64.4 (29/45)

Randomly selected clones were analyzed from (A) MTBLIB25 library (100–300 bp) and (B) MTBLIB27 library (300–800 bp) at various stages of library construction: 1, transformants obtained after large-scale electroporation of the ligation sample; 2, transductants obtained after infection of TOP10F’ cells with the primary phage library; 3, transductants obtained after infection of TOP10F’ cells with the trypsin-treated primary phages; 4, transductants obtained after infection of TOP10F’ cells with the secondary ORF-selected phage library.

* Percentage of recombinant clones that aligned to the *M. tuberculosis* (*M. tb*) genome.

† Percentage of clones in-frame with the PelB signal sequence (*PelBss*) and *gIIIP* in the phagemid.

‡ Percentage of total in-frame clones (as in III) that aligned with the *M. tb* proteome (genic ORFs).

Number of positive clones/total clones analyzed is given in brackets.

Analysis of C02 cells revealed that more than 90% of the clones (89.6% in the 100–300 bp library and 95.5% in the 300–800 bp library) contained in-frame inserts, and thereby carried ORF-selected DNA sequences (Table 2A and B, II.3). Of these, 50% clones contained sequences, which coded for proteins that aligned to the *M. tuberculosis* proteome (genic clones; Table 2A and B, III.3). This number was not surprising, as clones with in-frame non-genic inserts without stop codons would display a non-genic protein fused with trypsin-resistant gIIIP, even at the P01 stage. Phages derived from such clones would remain infectious upon trypsin treatment, and hence would be a part of C02 preparation. The large fragment (300–800 bp) library contained more genic clones than the small fragment library (100–300 bp). This is due to the fact that the likelihood of having a proper translatable frame without a stop codon in an alternate non-ORF reading frame is lower for larger fragments. Upon rescue with a helper phage, such as VCSM13 or even AGM13, each C02 clone was expected to produce phages carrying an in-frame insert, and display the encoded peptide fused to gIIIP. The phages were rescued from 2.5×10^9^ C02 cells using AGM13 to produce phage libraries named MTBLIB25P02 and MTBLIB27P02 for the 100–300 bp and 300–800 bp inserts, respectively. Analysis of P02 phages showed concordant results with those of C02, as more than 95% of the phages carried inserts in-frame with *PelBss* and *gIIIP*, and the proportion of genic clones was between 55–60% (Table 2A and B, III.4).

**Figure 5:**
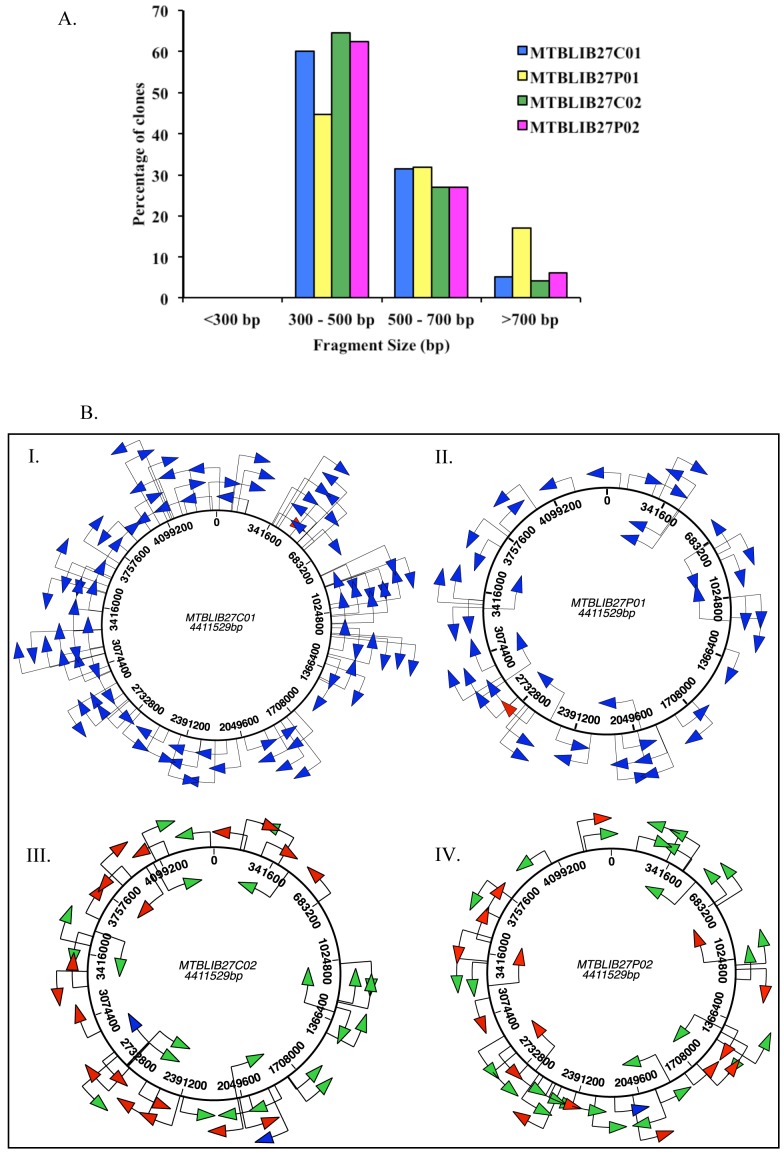
Distribution of *M. tuberculosis H37Rv* gene fragments in the MTBLIB27 library. **5A**. Bar graph depicting the size distribution of *M. tuberculosis H37Rv* gene fragments in the MTBLIB27 at different stages of library construction. **5B**. Schematic representation of the distribution of gene fragments. The *M. tuberculosis* genome is ∼4.4 Mb and consists of ∼4000 genes. (I) MTBLIB27C01 primary cells before ORF selection; (II) MTBLIB27P01 after trypsin treatment (10 µg/ml) of primary phages; (III) MTBLIB27C02 secondary cells obtained after trypsin treatment (10 µg/ml) of primary phages for ORF selection; (IV) MTBLIB27P02 obtained from rescue of C02. The non-ORF selected inserts which align with the *M. tuberculosis* genome are shown as blue arrows; the clones in-frame with *PelBss* and *gIIIP* are indicated as red arrows (non-genic clones); the clones aligning with the *M. tuberculosis* proteome are indicated as green arrows (genic clones). The direction of the arrows indicates gene orientation. The maps are to scale.

Another important feature of this ORF selection protocol is that the distribution of differently sized fragments was largely maintained (Fig. 5A; Table S2.A and B in Data S1) at every treatment and selection step, as more than 90% of the fragments in the MTBLIB25 library were between 100–300 bp. Similarly, more than 90% of the clones in the MTBLIB27 library carried inserts between 300–800 bp. Furthermore, alignment of the nucleotide and peptide sequences at every stage of the selection protocol (C01/P01/C02 or P02) showed that the clones in both libraries were randomly distributed across the *M. tuberculosis* genome, with no overlapping clones (after analyzing 48–96 clones), proving that the entire process did not introduce bias towards any particular region of the genome (Fig. 5B).

**Figure 6:**
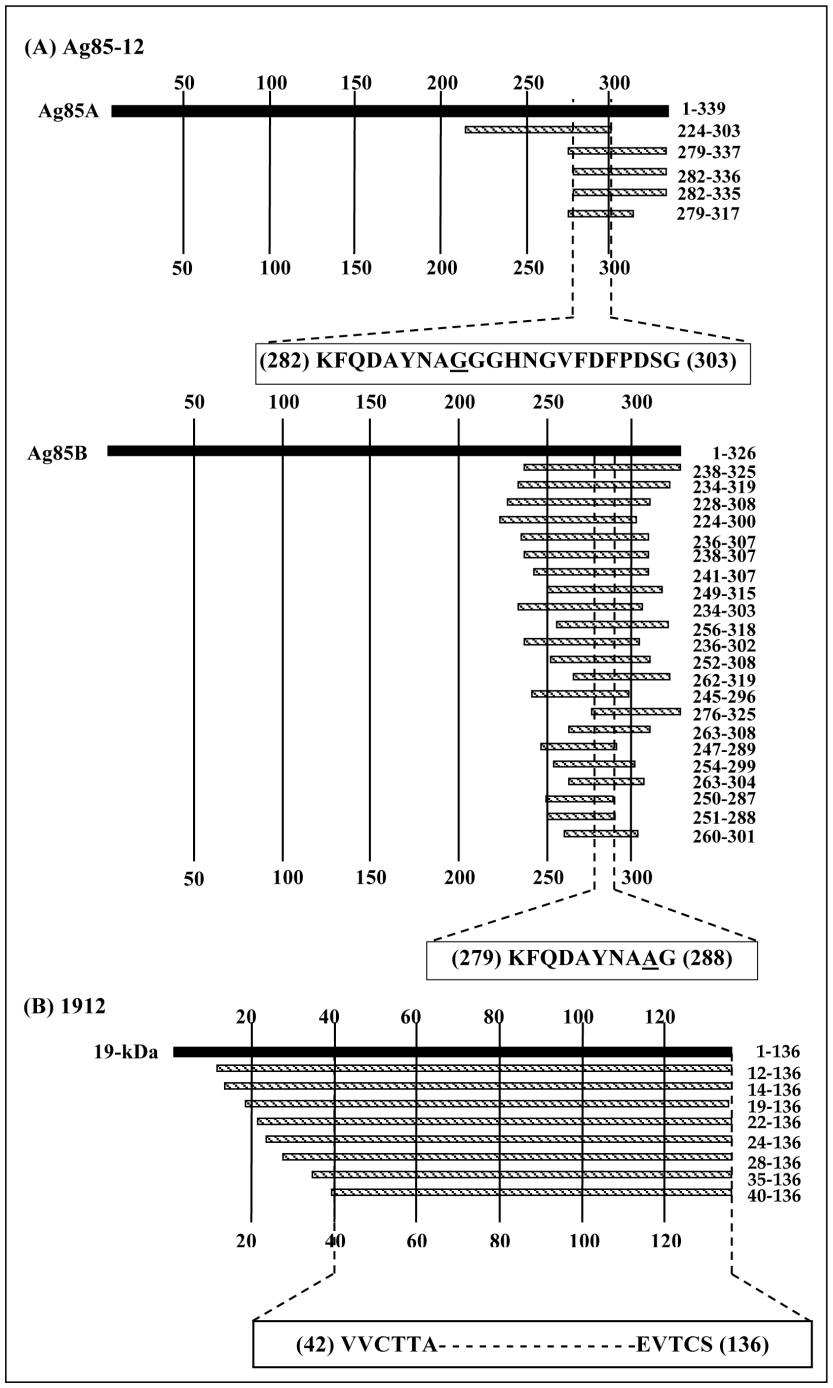
Alignment of the fragments of MTBLIB27 library selected by the monoclonal antibodies (A) Ag85-12 and (B) 1912. The translated regions of Ag85A, Ag85B and 19-kDa antigens of *M. tuberculosis* are represented as solid bar. The location of the deduced peptide sequences displayed on affinity-selected phages along with the position of the coded peptide is shown in bold on the right of the corresponding clone bar. The minimal overlapping sequence representing the putative epitope is shown below the alignment of each group.

ORF-selected phage libraries are expected to be several-fold more efficient than ORF-unselected libraries (P01). This was demonstrated by carrying out panning of these libraries on different monoclonal antibodies, MAb Ag8512, raised against mycobacterial antigens Ag85A or Ag85B, and MAb 1912, raised against 19 KDa protein. Due to high sequence similarity between Ag85A (Rv3804c) and Ag85B (Rv1886c), nearly 90% at amino acid level, MAb Ag8512 binds to both the proteins. Therefore, it was interesting to decipher if this MAb binds to the same sequence on Ag85A and Ag85B or to a sequence that is almost similar but has a few differences between Ag85A and Ag85B. Two rounds of panning with MTBLIB25 and MTBLIB27 were sufficient to select clones, which, upon DNA sequence analysis, aligned to a region of Ag85A and Ag85B. Of these, ∼78% of the clones (35 out of 45 analyzed) aligned to amino acid 224–325 of Ag85B (Table 3). A few clones carried inserts belonging to Ag85A in the region 217–327 amino acid residues. The overlapping sequenced region of various phage clones deduced that region between 279–288 amino acid residues (KFQDAYNA**A**G) of Ag85B might constitute the epitope recognized by Ag8512. The corresponding sequence in Ag85A aligned to 282–303 (KFQDAYNA**G**GGHNGVFDFPDSG) amino acid residues (Fig. 6) with a variation of only one residue (shown in bold). Since, these residues A or G have no side chains, their contribution in antibody binding might be minimal.

**Table 3:** Panning of whole genome fragment libraries on monoclonal antibodies raised against mycobacterial proteins.

Monoclonal Antibody	MAb Ag85-12						MAb 1912					
Rounds	Pan II				Pan III		Pan II				Pan III	
Library	MTBLIB25		MTBLIB27		MTBLIB27		MTBLIB25		MTBLIB27		MTBLIB27	
	Test	Control	Test	Control	Test	Control	Test	Control	Test	Control	Test	Control
Input phage	1×10^9^	1×10^9^	1×10^9^	1×10^9^	1×10^9^	1×10^9^	1×10^9^	1×10^9^	1×10^9^	1×10^9^	1×10^9^	1×10^9^
Output phage	3×10^6^	8×10^4^	3×10^5^	3×10^4^	1×10^6^	3×10^4^	1×10^6^	7×10^5^	9×10^5^	2×10^5^	1×10^6^	6×10^4^
Fold enrichment*	37.5		10.0		33.3		1.43		4.5		17	
% Specific clones (no. of positive/no. analyzed)† #	77.7 (35/45)		71.9 (23/32)		90.6 (29/32)		(0/47)		12.5 (4/32)		68.7 (22/32)	

Control and Test refer to wells coated with phosphate buffer saline (pH 7.2) and specific monoclonal antibody, respectively.

* Fold enrichment = output phage in test/output phage in control.

† The inserts in clones from test wells selected after panning were sequenced and aligned to the respective *M. tuberculosis* protein.

# All the clones analyzed were in frame with resepect to PelBss and gIIIP.

MTBLIB25 library failed to identify epitope recognized by MAb 1912 which binds to 19 KDa antigen (Rv3763) and is indicated to have an epitope comprising ∼100 amino acid residues that might be conformational in nature. Further, 19 KDa antigen used for producing MAb 1912 consists of 136 amino acids with two cysteine residues at 44^th^ and 135^th^ position of its protein sequence. This failure was attributed to the composition of this library as it contains none or a very few clones harboring inserts greater than 100 amino acid residues (∼300 bp). Therefore, epitope mapping of MAb 1912 was attempted using MTBLIB27 library that carries fragments of 300–800 bp encoding for more than 100 amino acids with maxima around 150–175 amino acids. After two rounds of panning on MAb 1912, 4 out of 32 screened clones aligned to the 19 KDa sequence with further enrichment up to 68% in third round of panning wherein majority of the clones (22 out of 32 screened) aligned to the 19 KDa sequence (Table 3). The alignment of different sequences obtained from the positive clones recognized a common sequence encompassing amino acid residues 42–136 of 19 KDa including two cysteine residues (Fig. 6).

A remarkable observation was that the library contained a large number of clones, which end at 136^th^ residue of 19 KDa antigen but have variable residues at the N-termini. It is very unlikely that the primary library (MTBLIB27P01) did not have clones with longer sequences beyond 136^th^ residue but such clones may have been eliminated due to stop codon after 136^th^ residue, last residue of 19 KDa, during the process of ORF selection. Therefore, the results described here clearly demonstrate that the technology illustrated here allows, construction of large gene fragment libraries of varying sizes with selection of ORF that can be efficiently employed for different applications.

In another example of functional antibody selection, AGM13 was used as a helper phage to rescue an anti-human red blood cell (anti-RBC) antibody phage display library. A mutant library (scFv) of 10^7^ clones (produced by mutagenesis using spiked oligonucleotides) for an anti-RBC antibody was constructed for the selection of improved binders, and the library was rescued using the helper phage AGM13. Phages produced from this unselected library (equivalent to P01) were screened for RBC binding using an agglutination assay [18]. Only 30% of the clones in the unselected library led to agglutination, indicating that only 30% of the clones expressed a functional scFv. Treatment of the primary library with trypsin led to complete elimination of the phagemid particles, which did not display full-length scFv-gIIIP fusion protein, to obtain a final enriched library wherein 100% of the clones produced phages that showed agglutination activity.

## Discussion

Phage display is a powerful technique for studying protein-ligand interactions. Despite the incredible success of antibody and peptide libraries, the use of phage display in cDNA libraries has been limited. This is related to the inefficient display of proteins and peptides, due to the occurrence of stop codons and poly(A) tails in cDNA, and insertion of the out-of-frame fragments, which means that only one of every 18 clones is capable of displaying a protein that could match with the proteome. For cDNA, the problem of stop codon and poly(A) tails can be overcome by using randomly primed libraries; however, the reading frame problem persists, which also hampers the use of gene fragments from a non-intron genome.

In this work, we designed a novel helper phage, AGM13, which was employed to produce genome-scale open reading frame (ORF)-enriched libraries of gene/genome fragments cloned into a gIIIP-based phage display vector. This unique application of AGM13 was made possible by the incorporation of trypsin cleavage sites within both of the flexible linker regions between the N1–N2 and N2-CT domains of gIIIP. As a result, the AGM13 helper phage encodes trypsin-sensitive gIIIP, and treatment with 10 µg/ml trypsin (∼0.42 µM) reduces phage infectivity by nine orders of magnitude. For ORF-enrichment applications, the foreign DNA to be displayed is cloned into the phagemid vector between the signal sequence and wild-type trypsin-resistant *gIIIP*. Furthermore, there is also a trypsin site located just before the first codon of wild-type *gIIIP* in the phagemid vector, which results in the conversion of phagemid encoded trypsin-resistant gIIIP fusion protein into trypsin-resistant gIIIP with full infectivity upon trypsin treatment. Thus, upon rescue of the phagemid-borne gene fragment library with AGM13, the in-frame clones encode trypsin-resistant gIIIP fusion protein, which becomes incorporated into the phages, along with other copies of helper phage AGM13-encoded trypsin-sensitive gIIIP. On the contrary, the clones harboring out-of-frame inserts produce phages carrying only trypsin-sensitive gIIIP. Treatment of the resulting phage library with trypsin renders the phages produced by clones with out-of-frame inserts non-infectious, whereas the phages rescued from clones with in-frame inserts remain fully infectious due to the presence of at least one copy of trypsin-resistant gIIIP. As a result, the transductants of a trypsin-treated library only carry clones with in-frame inserts and are thereby ORF-enriched.

The potential of AGM13 for ORF selection was evaluated by constructing two large whole genome fragment libraries (0.5–1×10^8^ clones) of *M. tuberculosis* (4.6 MB genome) with insert sizes of 100–300 and 300–800 bp. As expected, only one of every 18 clones in these randomly constructed libraries carried in-frame inserts to encode a peptide or protein, which could be displayed on the phage surface. The process of ORF selection by AGM13 comprises three simple steps (as depicted in Fig. 4): (i) production of phages (P01) from the transformants of the initial library in TOP10F’ cells (C01) using AGM13; (ii) trypsin treatment of the P01 phages and infection of TOP10F’ cells to obtain a library of ORF-selected transductants (C02); and (iii) rescue of phages from C02 using AGM13 or any other helper phage, such as M13K07 or VCSM13, to obtain ORF-selected phages displaying peptides/proteins (P02). These rescued phages can be directly employed for panning. The process of ORF selection described in this study is simple and enables nearly 100% enrichment of clones carrying in-frame inserts, irrespective of the size of the fragments. Further, approximately 60% of the clones corresponded to protein sequences, which aligned with the *M. tuberculosis* proteome (genic clones); the remainder were non-genic clones. The percentage of genic clones in the gene fragments would depend on the frequency of stop codons (TAA and TGA) in non-genic frame, which could vary with the GC content of a genome.

In the past, several different approaches have been explored to select in-frame ORFs for phage display of encoded peptides or proteins. One approach involves the expression of functional β-lactamase by clones carrying in-frame inserts to confer ampicillin resistance [12]. Here, the inserts must subsequently be made compatible with phage display, either by deleting the β-lactamase gene using recombination-based methods [12], or by transferring ORF-selected inserts using restriction enzyme-based cloning to phage display vectors [11]. This re-cloning step has been reported to reduce the incidence of ORF-selected clones to 70% from an initial selected library containing >95% ORF inserts. Another approach involves ORF-selection using Hyperphage (a helper phage with *ΔgIII*). Hyperphage-based ORF selection leads to enrichment of shorter-size inserts from a library containing longer inserts as well. Moreover the ORF-enrichment is relatively less efficient, enabling selection of only 69% of the clones carrying in-frame fragments [15]. The fusion of proteins at the N-terminus of gIIIP can influence the incorporation of the resulting fusion protein into phages during assembly, as well as the infectivity of the released phage particles if only the gIIIP fusion protein is displayed, as is the case with hyperphages [19]. Furthermore, phage assembly and infectivity can be adversely influenced by the size of the displayed protein, as clones with large inserts might generate fewer phages reducing their overall representation in the library. In the AGM13-based ORF selection strategy described in this paper, there is no size bias observed in ORF-enrichment and the same insert size distribution is observed in P01 and P02 libraries. This is because trypsin treatment of P01 phages (ORF-unselected primary phages) not only destroys the AGM13-encoded gIIIP, but also makes the trypsin-resistant gIIIP fusion protein fully infectious by removing the displayed peptide/protein. Thus, the transductants of ORF-selected phages (C02) are produced with equal efficiency, irrespective of the size of the inserts. Unlike the β-lactamase selection system, no re-cloning step is involved in the system described in this study.

AGM13-rescued phages offer additional advantages during affinity selection and elution of bound phages with trypsin as only specific binders would remain infectious after elution with trypsin, whereas non-specific phages would become non-infectious. This aspect has also been applied in the use of protease-sensitive helper phage KM13 in a previous study [2].

Numerous other methods of ORF selection have been reported for various applications; however, the majority is based on screening rather than selection and have inherent problems [20], [21]. T7 phage has been successfully employed for ORF selection in large libraries to obtain up to 90% ORF enrichment. However, the fragment sizes only ranged from 300 to 500 bp, which may be small to ensure representation of all protein domains [22]. The use of various solubility-based reporter systems such as chloramphenicol acetyltransferase (CAT) [23] or dihydrofolate reductase (DHFR) [24] can also be exploited for the purpose of ORF selection, but can face the problem of internal cryptic start sites within the gene fragments leading to expression of fusion proteins with the selectable marker in a non-genic reading frame. In this regard, insertion of random gene fragments between the signal sequence and β-lactamase gene has been used to efficiently select ORFs at a genome scale [25]. This method is predicted to allow the selection of gene fragments encoding soluble proteins. In contrast, the AGM13-based ORF enrichment technique described in this study eliminates internal start site clones and facilitates the generation of large and highly diverse ORF-selected phage libraries without any change in the average size distribution of the clones, and can be directly employed for affinity selection procedures to select binding ligands.

We also tested the utility of the AGM13 for antibody libraries wherein directed evolution of anti-RBC antibody was performed. Here, PCR-based spiked mutagenesis was used to create a library containing 10 million combinations for scFv. AGM13-based library production led to complete elimination of non-scFv displaying clones form the library.

The demonstrated use of AGM13 for genome-scale ORF selection is expected to facilitate the construction of randomly primed cDNA libraries in gIIIP-based M13 vectors. Such ORF-selected libraries could then be subcloned into an expression vector to produce the whole proteome in a test tube. These ORF-selected libraries could be transferred to an eukaryotic expression vector and be used for DNA immunization [26]. The technology of genome-wide ORF selection would be of immense value for the identification of vaccine candidates using techniques such as proteomic-based expression library screening [27] and *in vivo* induced antigen technology [IVIAT] [28], both of which currently require the cloning of randomly fragmented gene inserts into inducible expression vectors. After ORF selection with AGM13, the inserts could be transferred into yeast two-hybrid vectors for protein-protein interaction studies or to another phage display system for high-density display. Incidentally, the libraries used in this work have been constructed by incorporating features of non-enzymatic/recombinational transfer.

In conclusion, the helper phage AGM13 has equivalent properties to the current, most widely used helper phages, VCSM13. In addition, AGM13 enables the high efficiency selection of reading frames and therefore, AGM13 has a wide range of applications in the production of ORF-enriched phage displayed cDNA, gene-fragment and antibody libraries.

## Supporting Information

Data S1 **Figure S1.** Schematic representation of the vector pVCEPI23964. Only the relevant genes and restriction sites are shown. The map is not to scale. lacPO, lac promoter-operator; RBS, ribosome-binding site; PelB, pectate lyase signal sequence; Stuffer, 1.8 Kbp nucleotide sequence flanked by *Bsa*I sites (a) and (c); Tryp, trypsin protease cleavage site; S, spacer; gIIIP, segment encoding amino acid residues 2–405 of the gene III of filamentous phage; fori, origin of replication of filamentous phage; Amp^r^, β-lactamase gene; Ori, ColE1 origin of replication; tHP, transcriptional terminator. Details of regions marked a, b and c by double-headed arrows are shown in A, B and C, respectively. ‘D’ shows the sequence flanking the ORF after cloning into the phagemid vector harboring *att*B1 and *att*B2 site-specific recombination sites (in bold) based on Gateway Technology. Amino acids are shown in single-letter code below the nucleotide sequence. Restriction enzyme sites are shown above the nucleotide. **Figure S2.** **Western blot analysis.** Trypsin-untreated and -treated VCSM13 and AGM13 phages (1.5×10^10^) were separated by 10% SDS-PAGE under reducing conditions, transferred onto 0.45 µ PVDF membranes and probed with anti-gIIIP MAb 30421, which targets an epitope located in the N2 domain of gIIIP from the bacteriophage M13. M, Prestained marker; Lanes 1–5, 1.5×10^10^ VCSM13 phages treated with 0, 0.1, 1, 10 and 100 µg/ml trypsin. **Table S1. Analysis of random clones after treatment of primary phages (P01) with various concentrations of trypsin. Table S2. Size distribution of randomly sequenced clones at various stages of library construction.**
(PDF)
