# Supplementary material for: A Novel Helper Phage Enabling Construction of Genome-Scale ORF-Enriched Phage Display Libraries
Source: PLoS One. 2013 Sep 27;8(9):e75212. doi: 10.1371/journal.pone.0075212 (PMC3785514; doi:10.1371/journal.pone.0075212)
Supplement: Data S1 — Figure S1. Schematic representation of the vector pVCEPI23964. Only the relevant genes and restriction sites are shown. The map is not to scale. lacPO, lac promoter-operator; RBS, ribosome-binding site; PelB, pectate lyase signal sequence; Stuffer, 1.8 Kbp nucleotide sequence flanked by BsaI sites (a) and (c); Tryp, trypsin protease cleavage site; S, spacer; gIIIP, segment encoding amino acid residues 2–405 of the gene III of filamentous phage; fori, origin of replication of filamentous phage; Ampr, β-lactamase gene; Ori, ColE1 origin of replication; tHP, transcriptional terminator. Details of regions marked a, b and c by double-headed arrows are shown in A, B and C, respectively. ‘D’ shows the sequence flanking the ORF after cloning into the phagemid vector harboring attB1 and attB2 site-specific recombination sites (in bold) based on Gateway Technology. Amino acids are shown in single-letter code below the nucleotide sequence. Restriction enzyme sites are shown above the nucleotide. Figure S2. Western blot analysis. Trypsin-untreated and -treated VCSM13 and AGM13 phages (1.5×10^10^) were separated by 10% SDS-PAGE under reducing conditions, transferred onto 0.45 µ PVDF membranes and probed with anti-gIIIP MAb 30421, which targets an epitope located in the N2 domain of gIIIP from the bacteriophage M13. M, Prestained marker; Lanes 1–5, 1.5×10^10^ VCSM13 phages treated with 0, 0.1, 1, 10 and 100 µg/ml trypsin. Table S1. Analysis of random clones after treatment of primary phages (P01) with various concentrations of trypsin. Table S2. Size distribution of randomly sequenced clones at various stages of library construction. (PDF) [file pone.0075212.s001.pdf]

Table S1. Analysis of random clones after treatment of primary phages (P01) with various concentrations of trypsin.

Trypsin concentration	I. Fold reduction in titre	II. Aligning to *M. tb* genome	III. ORFs in-frame with *PelBss* and *gIIIp* *	IV. Aligning to the *M. tb* proteome †
**A. MTBLIB25 Library (100-300 bp)**				
Without trypsin	-	100 (32/32)	-	-
10 µg/ml	1 x 10^4	100 (32/32)	93.7 (30/32)	40.0 (12/30)
100 µg/ml	4 x 10^4	100 (32/32)	96.8 (31/32)	32.25 (10/31)
200 µg/ml	10^6	100 (32/32)	93.7 (30/32)	33.33 (10/30)
**B. MTBLIB27 Library (300-800 bp)**				
Without trypsin	-	100 (47/47)	-	-
10 µg/ml	1.3 x 10^4	100 (44/44)	100 (44/44)	65.9 (29/44)
100 µg/ml	1.7 x 10^4	100 (44/44)	93.2 (41/44)	53.6 (22/41)
200 µg/ml	10^5	97.7 (43/44)	95.4 (42/44)	57.1 (24/42)

Primary phages (10^{11}) from the (A) MTBLIB25 library (100-300 bp) and (B) MTBLIB27 library (300-800 bp) were incubated with four different concentrations of trypsin (0, 10, 100 and 200 µg/ml) and then used to infect *E. coli* TOP10F' cells to determine the reduction in phage titer. Randomly selected clones were analyzed by PCR and DNA sequencing to estimate the percentage of ORF (genic and non-genic ORF) and non-ORF clones.

* Percentage of clones in-frame with the *PelB* signal sequence and *gIIIp* in the phagemid.

† Percentage of total in-frame clones from Stage III that aligned with the *M. tuberculosis* (*M. tb*) proteome (genic clones).

Number of positive clones/total clones analyzed is given in brackets.

Table S2. Size distribution of randomly sequenced clones at various stages of library construction.

**A. MTBLIB25 Library (100-300 bp)**				
Size	1. MTBLIB25C01 (95)	2. MTBLIB25P01 (47)	3. MTBLIB25C02 (48)	4. MTBLIB25P02 (48)
< 100 bp	2	2	3	2
100 - 200 bp	42	25	24	29
200 - 300 bp	47	20	20	17
> 300 bp	1	-	1	-

**B. MTBLIB27 Library (300-800 bp)**				
Size	1. MTBLIB27C01 (95)	2. MTBLIB27P01 (47)	3. MTBLIB27C02 (48)	4. MTBLIB27P02 (48)
< 300 bp	-	-	-	-
300 - 400 bp	21	11	15	20
400 - 500 bp	36	10	16	10
500 - 600 bp	20	8	9	9
600 - 700 bp	10	7	4	4
700 - 800 bp	4	4	2	3
> 800 bp	1	4	-	-

The total number of clones analyzed at each stage are indicated in brackets. 1, Transformants obtained after large-scale electroporation of the ligation sample; 2, transductants obtained after infection of TOP10F' cells with the primary phage library; 3, transductants obtained after infection of TOP10F' cells with the trypsin-treated ORF phages; 4, transductants obtained after infection of TOP10F' cells with the secondary phage library.

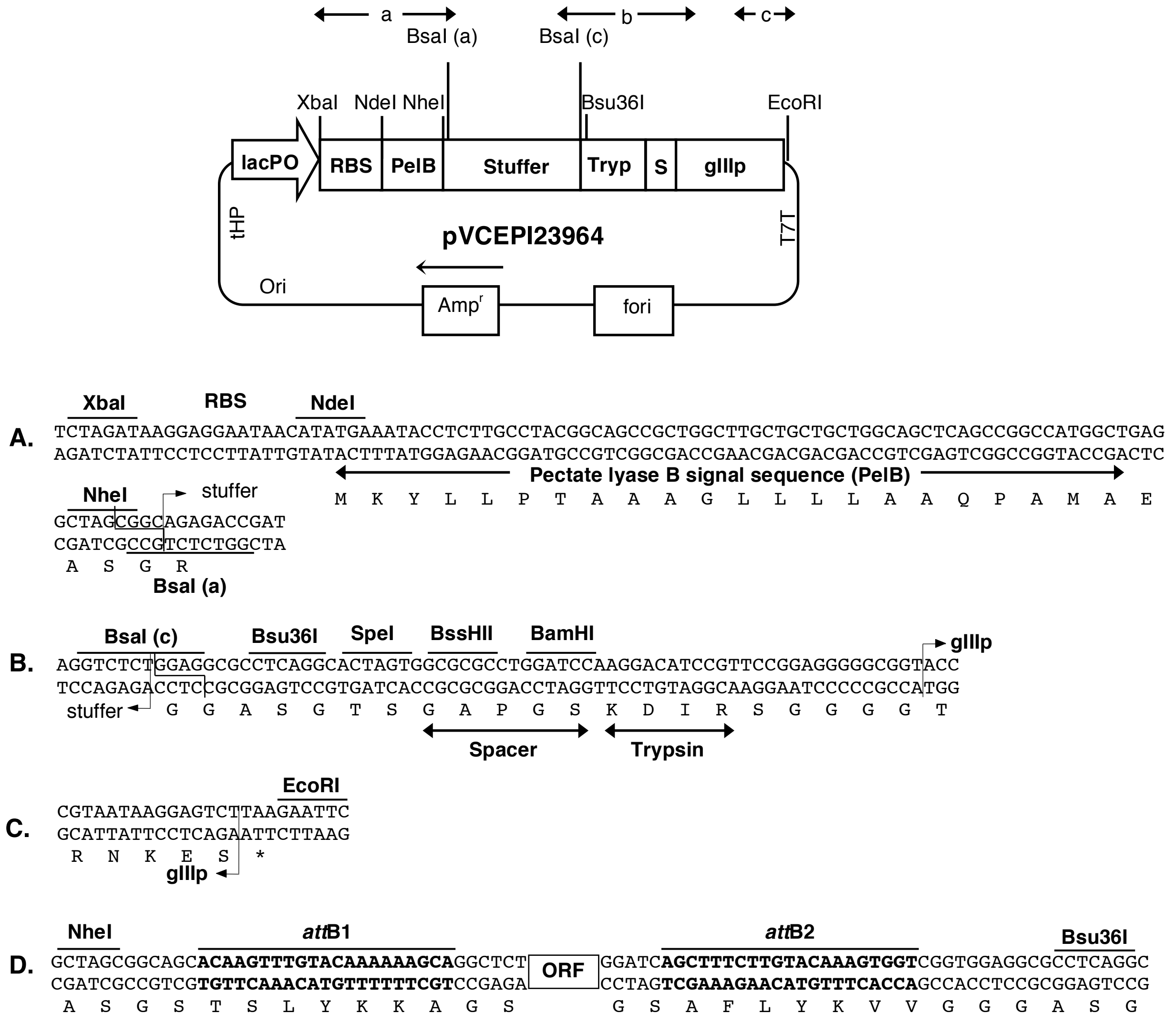

Fig. S1: Schematic representation of the vector pVCEPI23964.

Only the relevant genes and restriction sites are shown. The map is not to scale. lacPO, lac promoter-operator; RBS, ribosome-binding site; PelB, pectate lyase signal sequence; Stuffer, 1.8 Kbp nucleotide sequence flanked by *Bsa*I sites (a) and (c); Tryp, trypsin protease cleavage site; S, spacer; gIIIp, segment encoding amino acid residues 2 - 405 of the gene III of filamentous phage; fori, origin of replication of filamentous phage; Amp^r, β-lactamase gene; Ori, ColE1 origin of replication; tHP, transcriptional terminator. Details of regions marked a, b and c by double-headed arrows are shown in A, B and C, respectively. 'D' shows the sequence flanking the ORF after cloning into the phagemid vector harboring *att*B1 and *att*B2 site-specific recombination sites (in bold) based on Gateway Technology. Amino acids are shown in single-letter code below the nucleotide sequence. Restriction enzyme sites are shown above the nucleotide.

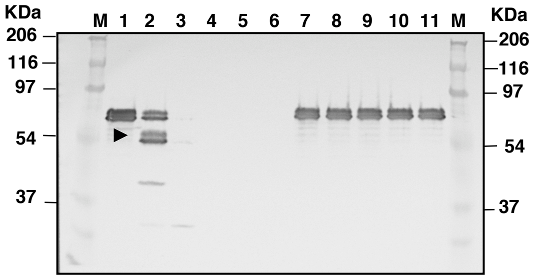

Fig. S2: Western blot analysis of helper phages.
Trypsin-untreated and -treated VCSM13 and AGM13 phages (1.5×10^{10}) were separated by 10% SDS-PAGE under reducing conditions, transferred onto 0.45 µ PVDF membranes and probed with anti-gIIIp MAb 30421, which targets an epitope located in the N2 domain of gIIIp from the bacteriophage M13. M, Prestained marker; Lane 1-5, 1.5×10^{10} VCSM13 phages treated with 0, 0.1, 1, 10 and 100 µg/ml trypsin, respectively; Lane 6, empty; Lane 7-11, 1.5×10^{10} AGM13 phages treated with 0, 0.1, 1, 10 and 100 µg/ml trypsin, respectively.
